# Snapshot multicolor fluorescence imaging using double multiplexing of excitation and emission on a single detector

**DOI:** 10.1038/s41598-021-99670-6

**Published:** 2021-10-14

**Authors:** Karolina Dorozynska, Simon Ek, Vassily Kornienko, David Andersson, Alexandra Andersson, Andreas Ehn, Elias Kristensson

**Affiliations:** 1 Department of Combustion Physics, Lund University, 22363 Lund, Sweden; 2 Department of Physical Chemistry, Lund University, 22100 Lund, Sweden

**Keywords:** Imaging and sensing, Fluorescence spectroscopy

## Abstract

Fluorescence-based multispectral imaging of rapidly moving or dynamic samples requires both fast two-dimensional data acquisition as well as sufficient spectral sensitivity for species separation. As the number of fluorophores in the experiment increases, meeting both these requirements becomes technically challenging. Although several solutions for fast imaging of multiple fluorophores exist, they all have one main restriction; they rely solely on spectrally resolving either the excitation- or the emission characteristics of the fluorophores. This inability directly limits how many fluorophores existing methods can simultaneously distinguish. Here we present a snapshot multispectral imaging approach that not only senses the excitation and emission characteristics of the probed fluorophores but also all cross term combinations of excitation and emission. To the best of the authors’ knowledge, this is the only snapshot multispectral imaging method that has this ability, allowing us to even sense and differentiate between light of equal wavelengths emitted from the same fluorescing species but where the signal components stem from different excitation sources. The current implementation of the technique allows us to simultaneously gather 24 different spectral images on a single detector, from which we demonstrate the ability to visualize and distinguish up to nine fluorophores within the visible wavelength range.

## Introduction

Fluorescence multispectral imaging is a powerful tool with applications in life science^1–4^, photonics^5–7^, engineering^8^ and drug discovery^9^. The technique uses fluorescent markers to determine concentrations, structures, functions and interactions between different sample components.

As the demand for information increases^2,5,6^, the diagnostic methods used require the means to increase the number of labels that can be simultaneously imaged. Traditionally, this is achieved using sequential imaging with one or several excitation wavelengths combined with optical filtering of the fluorescence signal^10^. In recent years, powerful scanning methods have been developed, such as the hyperspectral multicolor imaging reported by Jahr *et al.*, capable of distinguishing up to five fluorophores^7^. Another such example exploits multiplexed imaging based on stimulated Raman scattering^5,6^. In combination with engineered fluorescent probes whose spectral responses are well-separated, the visualization of a large number of distinctive species is enabled. However, both sequential and scanning approaches have a restricted temporal resolution and are therefore inefficient for the imaging of fast dynamic- or one-time events.

To meet the demand for imaging with higher temporal resolution, there has been an emergence of novel fluorescence-based fast, or even snapshot, multicolor imaging approaches^1,3,4,11–18^. In general, currently available solutions within this category are either based on sensing differences in the excitation spectra^1,3,4,12,15,17,18^ or by spectrally resolving the fluorescence emission^7,13,14,19^. For example, Mahou *et al.* demonstrated in 2012 a multicolor two-photon excitation-based imaging system capable of visualizing three chromophores simultaneously^18^. Lavagnino *et al.* constructed a snapshot hyperspectral emission-based imaging technique with 60 spectral channels, demonstrating the parallel determination of three fluorescence markers^19^. In 2018, Garbacik *et al.* developed a time-multiplexed excitation-based multispectral imaging system that further advanced the field, allowing for the visualization of six fluorophores, demonstrated for scanning confocal imaging^15^. Both excitation and emission-based approaches are, however, ultimately limited by (1) the spectral overlap between the different fluorophores, known as the ‘color barrier’, and (2) the unavoidable decrease in signal to noise ratio (SNR) in rapid/snapshot systems^20^. These limitations explain the restricted number of possible fluorescent markers within the visible/NIR spectrum that current technology can image simultaneously. However, even if many fluorescent markers may display large similarities in their excitation- or emission spectra, it is highly unlikely that *both* these characteristics are similar and hence being able to probe both properties simultaneously has the potential of greatly increasing the number of accessible fluorescent markers within the limited wavelength span offered by currently available cameras. To date, such multi-dimensional data can only be acquired using sequential/scanning imaging^5,6^ or time-multiplexing with multiple detectors^16,17^.

In this paper we report an alternative avenue for snapshot multispectral wide-field imaging that enables ‘full optical path tracing’, allowing us to sample both the excitation- and emission profiles of all fluorophores within a single acquisition and with a single detector. Unlike most existing configurations for multicolor imaging, our approach maintains the full temporal resolution of the detector and does not rely on any particular hardware, such as fast, time-resolved acquisition^4,15^ or pulsed lasers^5,6^. Instead, our configuration is based on the Frequency Recognition Algorithm for Multiple Exposures (FRAME) concept^21^, in which the excitation sources as well as the resulting fluorescence signals are spatially multiplexed, in two separate stages, yielding a doubly encoded intensity mapping of the sample. Using this novel approach alongside four different CW laser sources and four different spectral emission channels, we demonstrate that this combination yields a multiplexed image of 24 different images: one for each of the four lasers, one for each of the four spectral channels and 16 for all cross term combination. By analyzing these 24 images using linear unmixing, we demonstrate the ability to separate nine different fluorophores within the visible spectrum, despite significant overlap in their respective excitation and emission profiles. Furthermore, because we gain a more comprehensive and multi-dimensional picture of the fluorophores, we can achieve accurate distinction at a significantly lower SNR. To the best of the authors’ knowledge, this is the first demonstration of full optical path tracing-from source to detector-using multiple tandem illumination sources and spectral emission channels for snapshot imaging, providing high-throughput, low phototoxicity and outstanding spectral sensitivity.

## Results

### Double modulated FRAME

FRAME is a multiplexing imaging technique that allows several images—in this case carrying spectral information—to be acquired in parallel using a single detector^12,13,21^. With FRAME, multiplexing of the image data is achieved by superimposing a unique spatial frequency (intensity modulation) onto each individual image prior to detection. The captured image thus contains the sum of all intensity-modulated images and although the image information overlaps in the spatial domain, the data is separated in reciprocal space. By applying a frequency-sensitive lock-in detection algorithm the multiplexed image information can be extracted and separated, albeit at a reduced spatial resolution^21^. The spatial resolution of any FRAME-system is thus not directly dictated by the pixel resolution given by the camera, but rather by the distance between the imposed spatial frequencies in reciprocal space, which in turn sets the limit for the dimensions of the filter function used in the lock-in detection algorithm^22^. For the current optical setup, the spatial resolution is $\sim$ 3 lp/mm for the extracted frames and for a field-of-view of 21$\times$17 mm$^2$.

**Figure 1:**
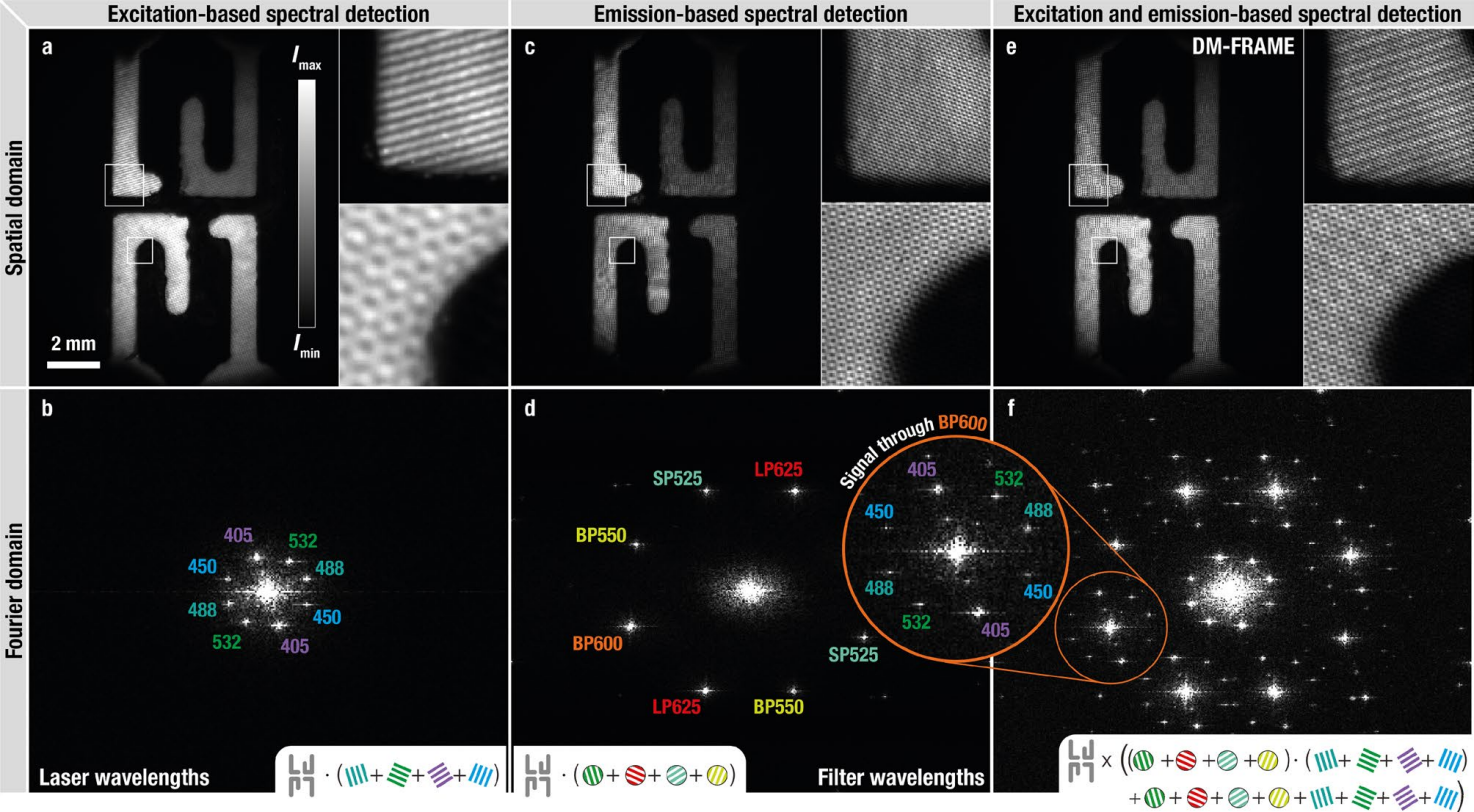
Example of DM-FRAME and its information storage capabilities. The fluorescence signals from four dyes are imaged using different spatial frequency multiplexing approaches. (**a**) The multispectral information is obtained by applying unique sine modulation patterns onto the intensity profile of four excitation light sources. (**b**) Fourier transform of (**a**). In (**c**) the multispectral information is obtained by guiding the light through four differently coded spectral emission channels (excitation at 450 nm). (**d**) Fourier transform of (**c**). (**e**) The two approaches combined; both the excitation sources and the detection channels are intensity-modulated. (**f**) Fourier transform of (**e**). The beating between the two applied intensity modulations yields satellite-like structures in the frequency domain. In total, this results in 24 pairs of peaks, each carrying unique 2D spectral information that can be accessed using a spatial frequency lock-in algorithm. SP = short-pass, BP = band-pass and LP = long-pass.

FRAME has previously been demonstrated to be capable of multispectral snapshot imaging using either excitation^12^ or emission-based^13^ image multiplexing, where the intensity-modulation encoding was applied on the different laser intensity profiles or the different spectral regions of the emitted fluorescence signal, respectively. Figure 1a–d, demonstrates the data acquisition for the respective cases. In this paper we demonstrate a combined approach, dubbed double modulated FRAME (DM-FRAME) where encoding is simultaneously applied to both the excitation and the emission. This approach allows us to sample and sense the molecular response (fluorescence) for each excitation wavelength of all fluorophores present in the sample within a single snapshot. This kind of cross term information, which previously only could be acquired using sequential or scanning-based imaging, is contained within the recorded image as beatings between the intensity-modulations of the excitation sources and those on the detection side. The beating results in a satellite-like structure in reciprocal space (Fig. 1f) where each such Fourier peak carries unique image information arising from a specific combination of excitation wavelength and optical filter. This spatial frequency-encoded spectral data is accessible by means of a spatial lock-in detection computer algorithm (Supplementary Information), which demodulates and isolates each Fourier component, in this case yielding 24 images, all with different spectroscopic information. However, only 16 of these 24 images contain truly unique information, setting the upper limit on the number of distinguishable fluorophores to 16 with the current optical configuration. As the spectral information is obtained using spatial encoding rather than e.g. dispersive elements, DM-FRAME has the unique ability to differentiate between fluorescence light emitted at exactly the same wavelength, simultaneously emitted by the same fluorophore but stemming from different excitation sources.

Here the DM-FRAME approach is demonstrated for fluorescence multicolor imaging, in particular to distinguish fluorophores that have strongly overlapping excitation- or emission characteristics within the visible wavelength region. Our results show that the more comprehensive and detailed view of the molecular response obtained using DM-FRAME leads to an improved species-specificity that, in turn, both increases the number of fluorophores that can be imaged simultaneously and reduces acquisition times beyond the current limitations.

### Unmixing fluorophores with highly overlapping spectral characteristics

**Figure 2:**
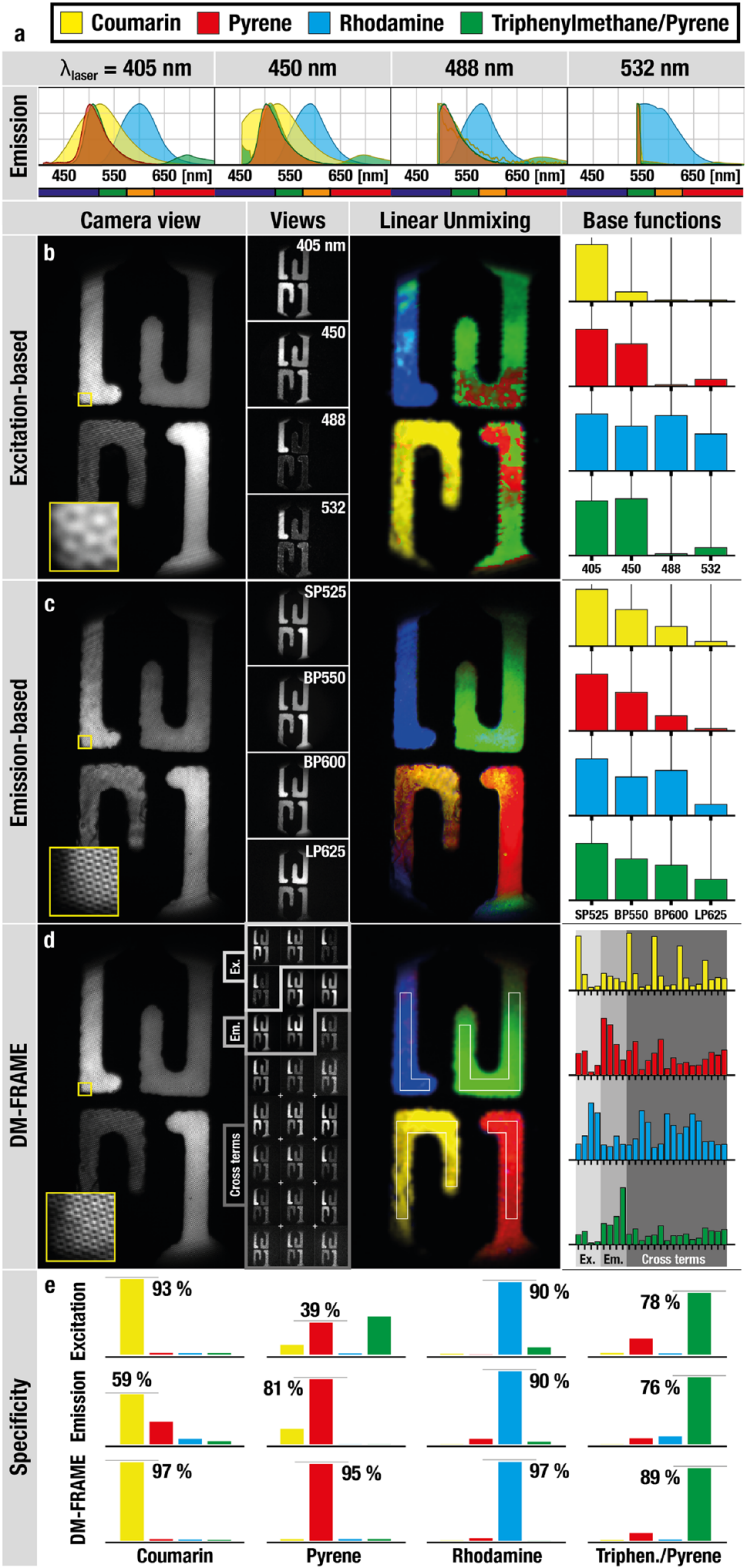
Improved spectral sensitivity gained by DM-FRAME. Measurements of four different dyes with very similar excitation and/or emission characteristics, analyzed using linear unmixing. (**a**) Spectral responses for each fluorophore at each excitation wavelength, together with an indication of the spectral bandwidths of the emission filters. (**b**) Excitation-based multiplexed imaging, (**c**) emission-based multiplexed imaging and (**d**) DM-FRAME. Each measurement contains sufficient information to theoretically identify the dyes but due to the strong spectral overlap, both the emission-based and excitation-based methods are prone to misidentification, see (**e**). However, when combining the methods using DM-FRAME, the faint differences between the dyes’ spectral characteristics can be detected, yielding highly accurate identification (measured for the signal in the areas marked in **d**).

**Figure 3:**
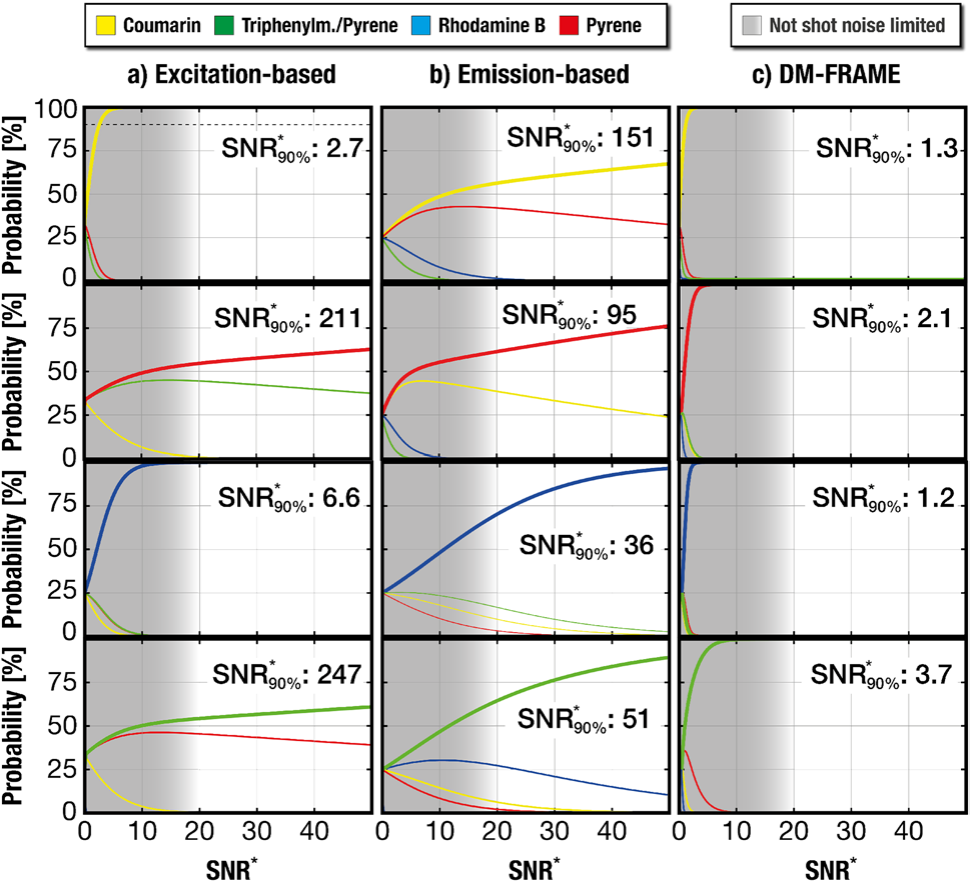
Estimations of the probability of correct fluorophore classification using the three different methods of Fig. 2, modeled for a purely shot noise limited system (indicated by the asterisk above the SNR). The indicated SNR* values correspond to an accurate classification of the fluorophores at a probability of 90%. (**a**, **b**). Neither the excitation- nor the emission-based detection scheme is able to accurately separate the full set of dyes at SNR* below 150. This susceptibility to noise induced uncertainties could hence be an explanation to the misidentifications observed in the linear unmixing of the corresponding cases in Fig. 2b,c, despite being mathematically solvable. (**c**) The ability of DM-FRAME to sense both emission- and excitation characteristics as well as the increased number of spectral channels it extracts yields a significantly improved species specificity, indicated by the fact the the curves reach 100% at low signal levels. Analysis of the background noise in our data indicates that the shot noise limited approximation holds for our imaging system at an SNR* above $\sim$15 (illustrated by the gray shadowing) and that the shot noise can be approximated by a Gaussian distribution^23^ above an SNR* $\sim$  5.

If an experiment only requires a few fluorescent labels it is often possible to select ones that have well-separated spectral excitation and/or emission signatures and thereby use suitable illumination sources or spectral filters to distinguish them^7^. However, the possibility to differentiate between fluorophores using such approaches diminishes as the demand for simultaneous imaging of a greater number of markers grows^4,5^. To demonstrate the improved species-specificity with our DM-FRAME method, four fluorophores (coumarin, rhodamine, pyrene and triphenylmethane/pyrene) with heavily overlapping spectral characteristics (Fig. 2a) were visualized using three different multispectral FRAME snapshot imaging configurations; (1) an excitation-based system with laser excitation at 405 nm, 450 nm, 488 nm and 532 nm, (2) an emission-based system equipped with optical filters, one shortpass ($<525\, \hbox {nm}$), two bandpass (525–575 nm and 575–625 nm) and one longpass ($>625\, \hbox {nm}$) and (3) a combination of these two systems, i.e. DM-FRAME. The results show that neither the excitation-based (Fig. 2b) nor the emission-based (Fig. 2c) systems are capable of accurately distinguishing the fluorophores through linear unmixing, even though the problem is mathematically solvable. This inability can be understood by examination of the similarities between the fluorescent responses-the base functions-of the individual fluorophores: pyrene and triphenylmethane/pyrene have an $R^2$-value of 0.98 for the excitation-based system whereas coumarin and pyrene have an $R^2$ value of 0.97 for the emission-based system. Distinguishing between fluorescent markers with such high $R^2$-values is feasible but requires high signal levels (Fig. 3a,b). Using the same optical components, i.e. the same excitation sources and optical filters, DM-FRAME overcomes the challenge of classifying markers with such similar spectral characteristics by extracting 24 different spectral images for each fluorophore using its unique full optical path tracing ability. Linear unmixing using these images (Fig. 2d) yields a 2D mapping of the four fluorophores with negligible misidentification and with a specificity that outperforms both excitation- and emission-based detection in all cases (Fig. 2e).

### Spectral unmixing of nine fluorophores

**Figure 4:**
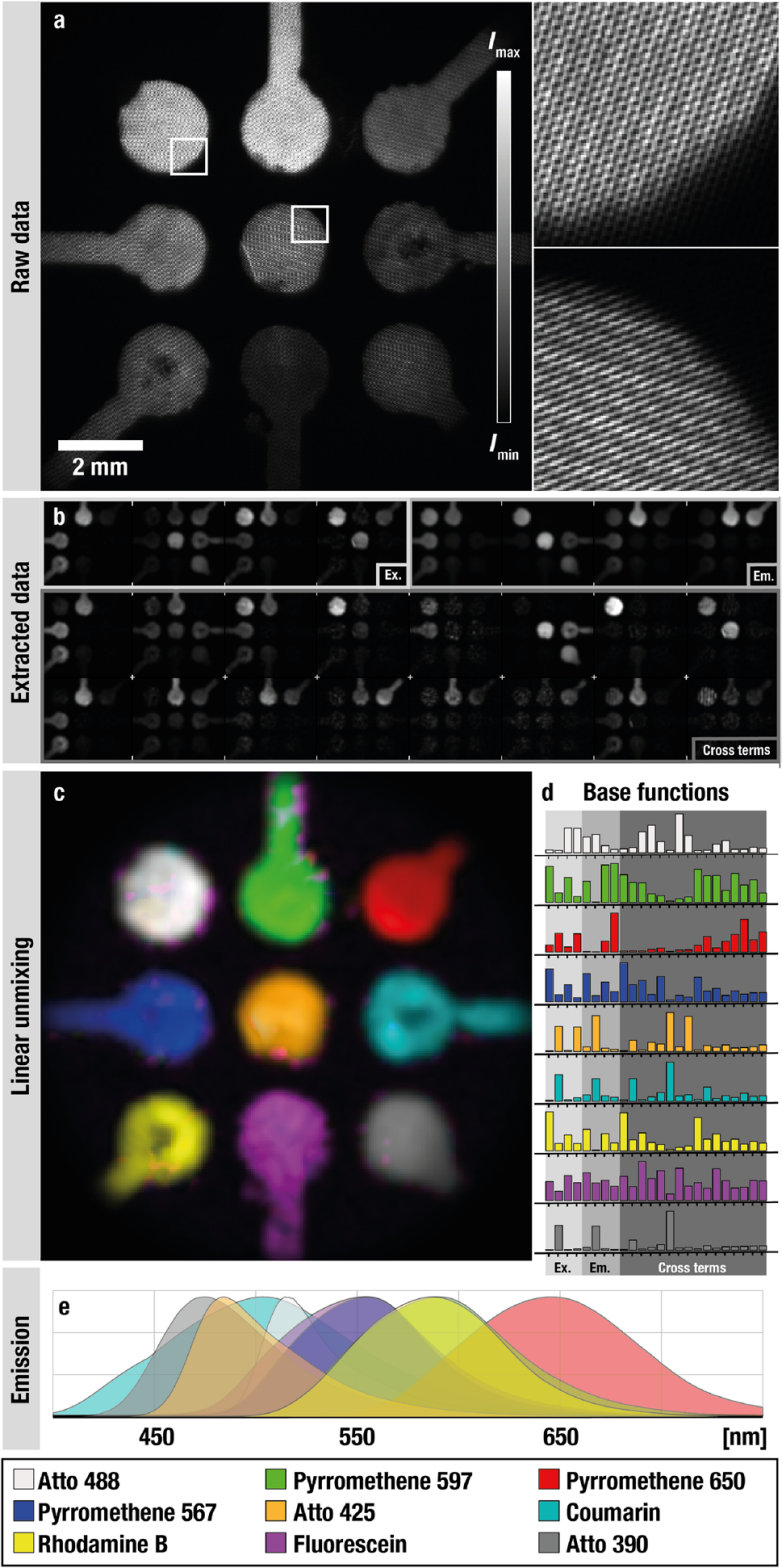
Linear unmixing of nine fluorophores. Nine fluorophores were probed using DM-FRAME where (**a**) is the raw unprocessed image as seen by the camera. The beating patterns, faintly visible in the magnified views, correspond to the doubly modulated information. (**b**) From the acquired data, 24 spectral images were extracted and subsequently used to perform the linear unmixing of the data. (**c**) The outcome of the spectral analysis, showing the nine different fluorophores in false colors. (**d**) The base functions (reference responses) for all nine fluorophores, where each fluorophore can be seen to acquire a unique footprint when imaged through the DM-FRAME system. (**e**) The spectrally resolved fluorescence signal (excitation 405 nm) for all nine fluorophores, indicating strong overlap of the featureless spectra.

Identifying a large number of fluorescent markers in a dynamic sample using multispectral imaging has been a long-standing challenge. Recent development towards fast hyperspectral imaging of multiple fluorophores are based on either scanning or sequential data acquisition, yet such approaches have a temporal resolution ultimately limited by the electronics or mechanical constraints^11^. The root challenge with simultaneous imaging of several fluorescent markers is that liquids and solids, unlike gas molecules, intrinsically have broad and featureless excitation- and emission profiles. In practice, it thus becomes difficult to find a range of suitable solid/liquid marker candidates that will not spectrally overlap significantly within the visible and near-IR region, where cameras are sensitive. Instead of attempting to circumvent the problem with broad excitation- and emission characteristics, DM-FRAME gathers a more comprehensive and complete view (24 spectral responses) of the probed markers, allowing us to fit a significantly higher number of fluorescent markers within the same camera-restricted wavelength span. To demonstrate this, we simultaneously image a sample containing nine different fluorophores (Fig. 4a) with highly overlapping spectral features (Fig. 4e) and successfully perform linear unmixing to distinguish them all (Fig. 4c). Since the number of fluorophores is higher than the number of excitation- and emission channels combined, access to the 16 cross-term responses (Fig. 4b and d) turn the otherwise underdetermined system of equations into an overdetermined one. To the best of the authors’ knowledge, this is the highest number of fluorescent markers simultaneously visualized using multispectral imaging.

### Multicolor imaging on a millisecond time scale

**Figure 5:**
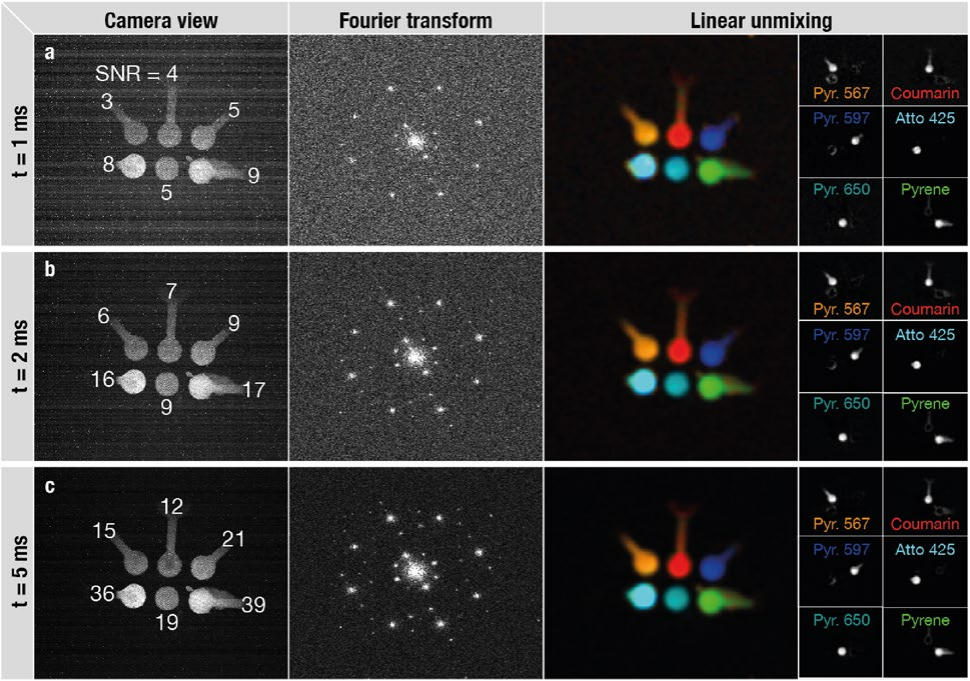
Multicolor imaging of six species on a millisecond time scale. Six species simultaneously visualized and separated using linear unmixing, with an acquisition time of (**a**) 1 ms, (**b**) 2 ms and (**c**) 5 ms. The results demonstrate the robustness of DM-FRAME and the ability to accurately separate fluorophores even at low SNR values (estimated SNR specified for each marker).

According to Cox and Sheppard, all imaging systems have a constant information capacity-bandwidth-that is, in turn, dictated by the system’s spatial and temporal parameters^24^. Any of these parameters can be increased beyond their classical limit or replaced by another type of data by reducing the bandwidth of another parameter. For example, super-resolution microscopy trades its temporal bandwidth for an augmented spatial bandwidth^25^. Similarly, several optical methods aiming at fast multicolor imaging of several fluorescent markers rely on time-based multiplexing^3–6,15^, thereby trading temporal resolution for access to spectroscopic data. For such approaches, the acquisition time unavoidably increases with the number of simultaneously measured markers, leading to a degraded temporal resolution. Emission-based methods have similar time constraints as these need to surpass a sufficient signal level in order to accurately differentiate the markers (see e.g. Fig. 4). Our solution-DM-FRAME-exploits the spatial domain to multiplex spectral data and we are therefore not bound by the same constraints as time-based methods, allowing us to record and separate several species simultaneously at record-breaking low integration times. We demonstrate this capability experimentally by (1) acquiring a multispectral 5.5 MPixel video at 4 Hz (Supplementary Video 1) and (2) recording and successfully de-multiplexing six species with an integration time of 1 millisecond (Fig. 5a). To the best of our knowledge, this is the fastest simultaneous multi-species acquisition of this many fluorophores to date.

A DM-FRAME configuration is highly adaptable and not restricted to a certain combination of excitation sources and optical filters. This versatility can be beneficial when probing a multiple number of fluorophores with low quantum yields as the excitation sources can be optimally selected so to provide maximum fluorescence emission. The results in Fig. 5, supported by the trends in Fig. 3, suggests that DM-FRAME is able to operate under such challenging, low signal conditions.

Finally, optimization of the detection configuration, i.e. replacing the beam splitters with more light-efficient dichroic mirrors, would yield a 16 fold increase in signal strength and thus a direct reduction in integration time, allowing for even faster data acquisition rates. Although the performance of DM-FRAME depends on e.g. pixel resolution and sample luminosity, its maximum acquisition rate is ultimately governed by the specifications of the camera system. It therefore holds potential for multicolor imaging of dynamic, one-time events in e.g. the kHz-MHz regime when combined with state-of-the-art high-speed video cameras^26^ or FRAME-based methods for videography^27^ that can offer high pixel resolutions.

## Discussion

**Figure 6:**
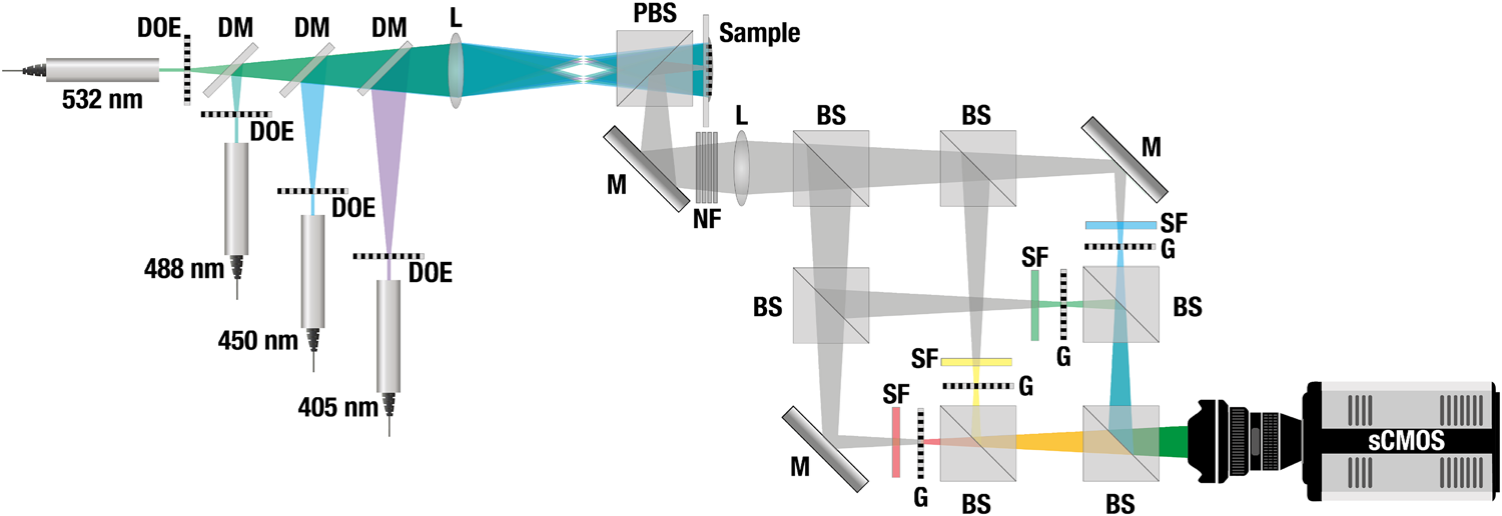
Schematic of the optical setup. The illumination consists of four CW laser sources, each guided through a diffractive optical element (DOE, company Holo/Or) which, when imaged onto the sample, creates a spatial intensity modulation pattern. The fluorescence emitted by the sample is then guided through a detection system where the light is split into four different paths. Each path contains a spectral filter (SF) and a transmission grating (G) which encodes the light. When the modulated fluorescence signal is transmitted through the gratings a unique beating pattern is created. This grants optical tracing abilities from the light source to the detector. DM = Dichroic Mirror, L = Lens, PBS = Polarizing Beam Splitter, M = Mirror, BS = Beam Splitter. NF = Notch Filter.

In summary, we have presented an optical arrangement-DM-FRAME-that allows the fluorescence of each probed fluorescent marker to be spectrally resolved in 2D for several different excitation wavelengths in parallel and with a single camera (illustration of the optical arrangement is given in Fig. 6). This feature allows the excitation of the fluorophores to be optimized, thereby providing high light-efficiency while simultaneously minimizing the risk for photobleaching. Technically, DM-FRAME performs two separate spatial multiplexing stages, one on the illumination side and one on the detection side. The acquired image is constructed by the product of these stages, i.e. a doubly multiplexed image. This technical advancement yields a powerful and effective means-full optical path tracing-to augment subtle differences in the spectral characteristics of different fluorescent markers otherwise only differentiable at high SNRs. Full optical path tracing thus opens up for multicolor imaging with high species-specificity using standard dyes and fluorescent markers. We demonstrate this capability here through the parallel detection of nine fluorophores-the highest number of simultaneously detected fluorophores to date-whose strongly overlapping fluorescence signals all reside within the visible wavelength region.

Full optical path tracing is accomplished by reducing the cameras spatial bandwidth. However, since the setup is all-optical, contains no moving parts and the data is acquired within a single snapshot, there are no direct requirements on the camera employed. The loss in spatial resolution can thus be partly remedied through either higher modulation frequencies on the illumination side^25^ or the use of modern high-resolution cameras, especially since only one detector is required. This lack of requirements further allows DM-FRAME to be combined with advanced intensified cameras for UV-sensitive multispectral detection or high-speed video cameras for high-speed multicolor videography in the future. In addition, the ability to perform temporally-resolved imaging may also be used to determine the fluorescence lifetimes^28,29^. Including such information in the data analysis together with the spectroscopic data provided by DM-FRAME, could potentially provide an even more enhanced species-specificity.

Furthermore, even if DM-FRAME is demonstrated for macroscopic epi-fluorescence in this paper, the technique is not bound to neither macroscopic imaging nor such a configuration but could, in principle, be adapted for all methods compatible with structured illumination, such as selective plane illumination^30^, two-photon excitation^31^ and total internal reflection imaging^32^.

Finally, fluorophore determination is not necessarily bound to imaging but could also be employed for e.g. screening and determination of protein concentration, where the parallelized detection provided by the DM-FRAME concept opens up for high-throughput spectroscopic analysis.

## Methods

### Optical setup

The experimental setup used consists of two encoding portions, illumination and emission encoding respectively. In the first encoding part, four continuous wave diode lasers operating at wavelengths of 405 nm (500 mW), 450 nm (2 W), 488 nm (120 mW) 532 nm (200 mW) are used. The lasers were chosen to be relatively evenly distributed over the more energetic part of the visible spectrum to enable excitation of the broad range of fluorophores used in the experiments. The lasers were further attenuated using ND-filters to match the power of the least powerful laser at 488 nm. The beams are initially expanded and collimated before propagating through individual diffractive optical elements (DOE), which splits each beam into two identical copies (angle between beams 0.4–0.6 degrees, corresponding to $\sim$ 10 lp/mm). The beams are then made collinear by spatially overlapping in a recombination arm, consisting of a series of dichroic mirrors (Thorlabs DMLP505R, DMLP425R and BSW10R). Using a lens the beam copies are made to overlap at the sample plane, where they interfere and produce a spatial intensity modulation pattern, according to their wavelength and relative angle. This modulated light is what illuminates the sample and due to the linear response the fluorescence emissions maintain the same encoding. Note that a non-linear response, such as that produced with two-photon excitation, would still produce the necessary fundamental Fourier components, although the approach would require a different calibration procedure. The emitted intensity-modulated fluorescence then enters the second part of the setup, where the emission encoding occurs. Notch filters are used at the entrance to the emission encoding setup in order to block any stray excitation light. The fluorescence emission is split equally, using beam splitters (Thorlabs BS031), into four optical paths. Each path contains a spectral filter (chosen for these measurements to be Thorlabs filters: shortpass 525 nm, bandpass 550 $\pm 25$ nm, bandpass 600 $\pm 25$ nm and longpass 625 nm) followed by a transmission Ronchi grating (Edmund Optics 2′′ $\times$ 2′′ Ronchi Ruling, 20 lp/mm), such that each different spectral emission range will have unique spatial modulation encoding. The emission filters were chosen to be evenly distributed over the entire visible spectrum. All the emissions are then recombined using beam splitters, before being imaged on to an Andor Zyla 5.5 sCMOS camera equipped with a 1$\times$ Edmund Optics GOLD-TL telecentric lens.

### The mathematics of a doubly modulated signal

FRAME, in any configuration, relies on applying a 2-dimensional intensity modulation, *M*, to an image signal:

1 $$\begin{aligned} M = \frac{1}{2}\big [\cos (\varvec{\omega }\mathbf {r} + \phi ) + 1\big ], \end{aligned}$$

where $\varvec{\omega } = (\omega _x,\omega _y)$ corresponds to the spatial frequencies in the x and y direction, and $\mathbf {r} = (x,y)$ corresponds to the two spatial dimensions of an image. When the modulated laser profile is incident on the fluorophores, the resulting response $I_f$ will consist of the underlying fluorophore response, *A*, superimposed with the laser modulation, $M_l$, such that:

2 $$\begin{aligned} I_f = AM_l = A\frac{1}{2}\big (\cos (\varvec{\omega }_{\mathbf{l}}\mathbf {r} + \phi _l) + 1\big ). \end{aligned}$$

$\varvec{\omega }_{\mathbf{l}}$ is the modulation frequency of the laser profile with an unknown phase $\phi _l$. In DM-FRAME, this fluorescence response, $I_f$, travels through the imaging system, via spectral filters, to then be superimposed with another spatial modulation of frequency $\varvec{\omega }_{\mathbf{e}}$ and phase $\phi _e$, resulting in a total signal of the form:

$$\begin{aligned} I= & {} \tilde{A}M_l M_e = \tilde{A} \frac{1}{2}\big (\cos (\varvec{\omega }_{\mathbf{l}}\mathbf {r} + \phi _l) + 1\big ) \times \frac{1}{2}\big (\cos (\varvec{\omega }_{\mathbf{e}}\mathbf {r} + \phi _e) + 1\big ), \end{aligned}$$

where $\tilde{A}$ is the spectrally filtered response. In the configuration presented in the manuscript, *I* corresponds to the final signal that is imaged on the sensor (e.g. Fig. 1a, c, e). Expanding the brackets and applying a product-to-sum trigonometric identity, *I* can be written as a sum of cosine terms:

$$\begin{aligned} I = \frac{1}{4}\tilde{A}\bigg ( \cos \big [(\varvec{\omega }_{\mathbf{l}} + \varvec{\omega }_{\mathbf{e}})\mathbf {r} + \phi ^+\big ] + \cos \big [(\varvec{\omega }_{\mathbf{l}} - \varvec{\omega }_{\mathbf{e}})\mathbf {r} + \phi ^-\big ] \nonumber + \cos ({{\varvec{\omega }}_{\mathbf{l r}}} + \phi _l) + \cos ({{\varvec{\omega }}_{\mathbf{er}} + \phi _e}) \bigg ). \end{aligned}$$

Hence the image will consist of the underlying fluorophore distribution not only modulated by the base frequencies applied by the excitation and emission, $\varvec{\omega }_{\mathbf{l}}$ and $\varvec{\omega }_{\mathbf{e}}$, but also their sum and difference: ($\varvec{\omega }_{\mathbf{l}} + \varvec{\omega }_{\mathbf{e}}$), ($\varvec{\omega }_{\mathbf{l}} - \varvec{\omega }_{\mathbf{e}})$. Performing spatial lock-in detection^21^ on the emission or excitation frequency components (the $\varvec{\omega }_{\mathbf{l}}$ and $\varvec{\omega }_{\mathbf{e}}$ terms) results in images of the total excitation/emission profile while performing the lock-in detection on the frequency-sum components will reveal the fully optically traced information about the fluorophore distribution, i.e. which lasers cause the fluorophores to emit which signal. Examples of all the extracted images can for example be seen in the views of Fig. 2.

### Linear unmixing

Linear unmixing is a mathematical approach^33,34^, used in fluorescence imaging, for identifying the different fluorophores, as well as their relative quantities, present in a sample. Linear unmixing requires calibration, or reference spectra, for each of the different fluorophores that the sample may contain. These reference spectra contain the response of each fluorophore for a given laser excitation and spectral filter. The greater the difference in response, the greater the sensitivity becomes and it is therefore beneficial to select fluorophores that either have fluorescence in different spectral regions or that have large differences in their excitation spectra. These reference spectra are then used to solve a series of simultaneous equations, which represent the captured spectrum. This can be done using a least squares fitting method which aims to minimize the square of the difference between the calculated and measured values. As a result the relative concentrations of each fluorophore in the mixed signal, can be determined.

## Supplementary Information

Supplementary Information 1.

Supplementary Information 2.
