# Supplementary material for: Snapshot multicolor fluorescence imaging using double multiplexing of excitation and emission on a single detector
Source: Sci Rep. 2021 Oct 14;11:20454. doi: 10.1038/s41598-021-99670-6 (PMC8517015; doi:10.1038/s41598-021-99670-6)
Supplement: Supplementary file 1 — Supplementary Information 1. [file 41598_2021_99670_MOESM1_ESM.pdf]

Snapshot multicolor fluorescence imaging using double multiplexing of excitation and emission on a single detector

Karolina Dorozynska[1], Simon Ek[1], Vassily Kornienko[1], David Andersson[1], Alexandra Andersson[2], Andreas Ehn[1], and Elias Kristensson[1]

[1]Lund University, Department of Combustion Physics, Lund, 22363, Sweden.
[2]Lund University, Department of Physical Chemistry, Lund, 22100, Sweden.
[*]elias.kristensson@forbrf.lth.se

Supplementary Information

Probability of classification

Given a set of n classes $C = \{C_1, C_2, ...C_n\}$, where each class C_k has m features $x_k = (x_1, x_2, ...x_m)$, the probability that a certain class $C_{k'}$ is being probed, given that a certain set of features, $\tilde{x_k}$, is measured, is given by Bayes theorem:

$$P(C_{k'}|\tilde{x_k}) = \frac{P(C_{k'})P(\tilde{x_k}|C_{k'})}{P(\tilde{x_k})}. \tag{1}$$

In other words, this gives the *a posteriori* probability of identifying a certain class, C'_k, given that a certain set of features, $\tilde{x_k}$, has been measured.

The aim of this section is to apply this to the identification of fluorophores, where C_k is the class of fluorophore, $C_k = \{\text{yellow}, \text{blue}, \text{green}, \text{red}\}$, x_k is the set of features corresponding to a certain class, i.e. the ground truth spectral signals obtained from a noise-free measurement of a given fluorophore, and $\tilde{x_k}$ is a noisy measurement performed on the fluorophore with our system.

Building the vector spaces

A class k' corresponds to the ground truth response of a given fluorophore that has been imaged through a FRAME system, either based on structured detection, structured illumination or the combination of the two. The different techniques divide the total relevant wavelength range into a set of bases, and the response of a given fluorophore is given by the coordinates in a multidimensional space spanned by said bases. In other words, each set of bases, created by either laser wavelengths, spectral filters or their combinations span a multi dimensional vector space, and the amount of light emitted by the fluorophore detected through the given basis system is given by the coordinates in this space. For structured detection the bases correspond to the wavelength profiles of the filters, while for structured illumination the bases correspond to the laser wavelengths. Hence, mathematically, the *emission* and *excitation* vector spaces, spanned by the filter characteristics (f) and laser characteristics (l) are given by:

$$\textbf{emission} = \text{span}\{\hat{f}_1, \hat{f}_2, \hat{f}_3, \hat{f}_4\} \quad \textbf{excitation} = \text{span}\{\hat{l}_1, \hat{l}_2, \hat{l}_3, \hat{l}_4\}$$

where:

$$\begin{cases} \hat{f}_i \cdot \hat{f}_j = 0 \text{ if } i \neq j \\ \hat{f}_i \cdot \hat{f}_j = 1 \text{ if } i = j \end{cases} \qquad \begin{cases} \hat{l}_i \cdot \hat{l}_j = 0 \text{ if } i \neq j \\ \hat{l}_i \cdot \hat{l}_j = 1 \text{ if } i = j \end{cases}$$

The orthonormality condition for these sets hold even though they do not strictly need to be orthogonal for the linear unmixing algorithm to funciton. Indeed they are easy to normalize by integrating wrt the wavelength, and equating to one and the wavelength range for the filters and lasers do not overlap, thus ensuring orthogonality.

Representing fluorophores as coordinates in these vector spaces

In these two basis sets, **emission** and **excitation**, a given fluorophore response can be described as coordinates within these vector spaces. So for example, fluorophore C_{blue} can be described with scalars $\{b_{f1}, b_{f2}, b_{f3}, b_{f4}\}$ s.t. its response, x'_{blue}, is given by:

$$x'_{\text{blue}} = b_{f1}\hat{f}_1 + b_{f2}\hat{f}_2 + b_{f3}\hat{f}_3 + b_{f4}\hat{f}_4, \tag{2}$$

x as seen through the **emission** basis. Equivalently for the **excitation** basis:

$$x'_{\text{blue}} = b_{l1}\hat{l}_1 + b_{l2}\hat{l}_2 + b_{l3}\hat{l}_3 + b_{l4}\hat{l}_4. \tag{3}$$

The values b are said to be the features of the class C_{blue}, given a certain measurement approach. They are experimentally given as the values of the bar plots of Fig. 2 in the main manuscript.

Double Modulation vector space

When using double modulation, we create a new vector space including that of the emission- and excitation spaces as well as their cross-terms. Hence it has the basis set:

$$\text{DM} = \text{span}\{\hat{f}_1, ..., \hat{f}_4, \hat{l}_1, ..., \hat{l}_4, \widehat{f_1 l_1}, \widehat{f_1 l_2}, ..., \widehat{f_4 l_4}\}. \tag{4}$$

The double modulation vector space is a 24 dimensional vector space where each fluorophore is described by 24 scalars/features. This is a mathematical explanation as to why the sensitivity of DM-FRAME seems at first glance to be better than simply excitation or emission based FRAME - we are describing each fluorophore with many more dimensions. Intuitively this makes sense as each image gives information about the emission intensity through a certain spectral filter bandwidth, excited with a certain laser, i.e. full optical path tracing.

Signal to noise ratio

The difference between a certain class's ground truth features, x_k, and a real measurement of said features is that the measurement suffers from noise. Hence we create an artificial measurement, $\tilde{x}_k$, where we simply add a noise component to the ground truth. The aim is to look at how well this noisy measurement can be identified as the correct fluorophore class using the different methods; structured illumination, structured detection and and the combination, i.e. double modulation. We assume the noise to be normally distributed random noise with expectation value 0. Then, we set the variance of the noise distribution, given a signal power s, as:

$$\sigma^2 = \frac{s}{\text{SNR}}. \tag{5}$$

In this context, the scalar values of eq 2 and 3 (the value of the class features) are the corresponding signal powers, s.

Classification

As described earlier, the aim is to look into the a posteriori probability that a certain noisy measurement, $\tilde{x}$, results from probing the ground truth features of a certain class, x_k: $P(C'_k|\tilde{x})$.

It is actually easier to flip the problem (as is shown in Bayes theorem, eq. 1) by asking: given the variance of the coordinates/features within measurement $\tilde{x}$, what is the probability that the ground truth values of x_k lie within this distribution. This can be evaluated by extracting the probability density function (pdf) of $\tilde{x}$, evaluated at ground truth class features x_k. Normalizing such that the pdfs add up to unity, will give the probability that a given noisy measurement is identified as a certain fluorophore.

The calculated a posteriori probability for all measurements into all fluorophore classes as a function of SNR was performed and is shown in Fig. 3 in the main text. The different colours of the graph correspond to the different fluorophores, color coded as in the main text. Each row corresponds to the probability of classifying the fluorophores as yellow, red, blue and green in that order. At infinite SNR (zero noise variance) one can see that the correct fluorophore tends to a probability of one, as expected. The columns correspond to the different measurement methods, which mathematically speaking, span the wavelength range with filters, lasers or the combination of the two.

Classification thresholds

Threshold SNRs were extracted from the datasets of Fig. 3 in the main manuscript, where the SNR that allows for a correct discrimination of fluorophores with a 90% and 95% probability are displayed in the table below.

Limitations of a signal dependent, Gaussian shot noise limited system

The above analysis is performed for a system where shot noise originating from the signal is assumed to be dominant. In order for signal dependent shot noise to be the main noise source in an imaging system, one must surpass a certain signal level set by the characteristics of the measurement system. Based on the background noise levels present in our data, we estimate this limit to be at an SNR of $\sim$15 above which the shot noise approximation holds. At SNR values below 15, however, the noise in the system becomes more complex with additional factors to consider (e.g. thermal- and readout noise). In addition, below an SNR of 5, a Gaussian approximation of the Poisson distributed shot noise does not hold any longer[23]. However, the main trend of a more accurate fluorophore classification when using DM-FRAME compared to solely emission- or excitation based FRAME can be seen for SNR values well beyond these model limitations, where neither emission- nor excitation based FRAME is able to classify the four fluorophores with a 90% probability below an SNR of 150.

Supp. Table 1. Low necessary SNRs for correct classification using DM-FRAME. The SNR of excitation-, emission- and DM-FRAME have been extracted where the probability of correct classification is at a threshold of 90 and 95% respectively. Again, high SNRs are needed for the individual methods due to highly overlapping fluorophore spectra.

		Coumarin	Pyrene	Rhodamine B	Trimethylm./Pyrene
**SNR_{ex}**	90%	2.7	210.9	6.6	246.8
	95%	3.4	282.6	8.1	330.9
**SNR_{em}**	90%	150.9	94.7	35.6	51.5
	95%	202.1	126.0	44.9	68.4
**SNR_{DM}**	90%	1.3	2.1	1.2	3.7
	95%	1.7	2.6	1.5	4.9

Base functions for DM-FRAME

Supp. Table 2. Base function values for the DM-FRAME case presented in Fig. 2. All base functions have an integrated value of unity.

	Coumarin	Pyrene	Rhodamine B	Trimethylm./Pyrene
**Excitation**	0.1303	0.0434	0.0242	0.0306
	0.0375	0.0483	0.0368	0.0410
	0.0067	0.0066	0.0869	0.0053
	0.0093	0.0198	0.0718	0.0091
**Emission**	0.0403	0.1138	0.0229	0.0653
	0.0333	0.1007	0.0206	0.0603
	0.0280	0.0614	0.0363	0.0875
	0.0205	0.0302	0.0237	0.1763
**Cross terms**	0.1370	0.0489	0.0227	0.0320
	0.0392	0.0670	0.0321	0.0483
	0.0072	0.0112	0.0752	0.0132
	0.0117	0.0291	0.0574	0.0259
	0.1294	0.0442	0.0187	0.0294
	0.0337	0.0718	0.0242	0.0581
	0.0099	0.0135	0.0705	0.0125
	0.0130	0.0346	0.0526	0.0272
	0.1063	0.0224	0.0402	0.0274
	0.0291	0.0258	0.0504	0.0331
	0.0097	0.0191	0.0704	0.0154
	0.0141	0.0201	0.0594	0.0201
	0.0713	0.0321	0.0277	0.0516
	0.0245	0.0459	0.0283	0.0429
	0.0302	0.0399	0.0230	0.0409
	0.0278	0.0503	0.0238	0.0467
